# Acute inflammation elicits decreased blood pressure but similar arterial stiffness in young African American adults

**DOI:** 10.1113/EP091289

**Published:** 2023-12-04

**Authors:** Lauren E. Eagan, Sara E. Mascone, Catalina A. Chesney, Sushant M. Ranadive

**Affiliations:** ^1^ Department of Kinesiology, School of Public Health University of Maryland College Park Maryland USA

**Keywords:** interleukin‐6, pulse wave velocity, racial differences

## Abstract

African Americans (AA) have a higher risk for cardiovascular disease (CVD) as compared to their White (W) counterparts. CVD is characterized by increased blood pressure (BP), arterial stiffness and systemic inflammation. An acute inflammatory stimulus may explain physiological manifestations responsible for amplified CVD in AA that are not apparent at rest. The purpose of this study was to evaluate central and peripheral BP, central and local arterial stiffness, and indices of pulse wave morphology in young healthy AA and W participants in response to acute inflammation. Concentrations of the inflammatory cytokine interleukin‐6 (IL‐6) and measures of central and peripheral BP, central arterial stiffness (carotid–femoral pulse wave velocity (cfPWV)), local carotid arterial stiffness (β‐stiffness, elastic modulus (Ep)), and indices of pulse wave morphology were assessed in 28 participants (21 ± 2 years, AA: *n =* 11) at baseline (BL), 24 h and 48 h post‐inflammation. Changes in IL‐6 concentrations (ΔIL‐6) were significantly greater at 24 h as compared to 48 h post‐inflammation (0.652 ± 0.644 vs. −0.146 ± 0.532 pg/μl, *P* ≤ 0.0001). Among AA participants, central and peripheral diastolic BP were significantly decreased at 24 h post‐inflammation as compared to BL (aortic diastolic BP: −4 ± 4 mmHg, *P* = 0.016; brachial diastolic BP: −4 ± 4 mmHg, *P* = 0.014). AA participants also experienced a significant decrease in central and peripheral mean arterial BP at 48 h post‐inflammation as compared to BL (aortic mean arterial pressure: −4 ± 4 mmHg, *P* = 0.009; brachial mean arterial pressure: −4 ± 4 mmHg, *P* = 0.012). Despite haemodynamic changes, there were no differences in central or local carotid arterial stiffness or indices of pulse wave morphology.

## Abstract (highlights)

- **What is the central question of this study?**
  Are there differences in interleukin‐6 concentrations, blood pressure, arterial stiffness and indices of pulse wave morphology between young healthy African American (AA) and White (W) individuals in response to an acute inflammatory stimulus?
- **What is the main finding and its importance?**
  While young healthy AA and W adults have similar interleukin‐6 concentrations, arterial stiffness and indices of pulse wave morphology in response to an acute inflammatory stimulus, only AA experience decreases in blood pressure within 48 h post‐stimulus. The findings could elucidate physiological manifestations responsible for amplified CVD in AA.

## 1 INTRODUCTION

Cardiovascular disease (CVD) remains the leading cause of death among all adults in the USA, yet the prevalence and incidence of CVD are notably higher among African American (AA) adults as compared to their White (W) counterparts (Tsao et al., 2023). While the causes of CVD are multifactorial, underlying impairments contributing to higher risk for CVD have been shown to manifest as early as the second decade of life and can be detected before any overt signs or symptoms are present (Heffernan et al., 2008; Tsao et al., 2023).

Underlying cardiovascular dysfunction is typically characterized by weakened vasodilatory capacity, increased central and peripheral blood pressure (BP), reduced vascular tone, and stiffening of the central and/or peripheral arteries (Vlachopoulos et al., 2010). As BP waveforms propagate from the central arteries to the peripheral arteries, they generate ‘forward traveling’ momentum. As the forward wave is reflected from various bifurcations, including the resistance arteries, a backward wave is generated towards the aorta. Stiffer or vasoconstricted arteries propagate the reflected waves faster, eventually causing augmentation of the systolic pressure. This occurrence can be quantified using the augmentation index (AIx) (Laurent et al., 2006). Therefore, higher AIx values correspond with stiffer and/or vasoconstricted arteries, increased blood pressure and thus greater CVD risk (Davies & Struthers, 2005).

Along these lines, some (Ashraf et al., 2012; Heffernan et al., 2008, 2007; Lefferts et al., 2017; Liang et al., 2019) but not all (Heffernan et al., 2009; Yan et al., 2013, 2016) of the existing literature suggests that at rest, young healthy AA individuals have heightened indices of arterial stiffness and AIx as compared to their W counterparts – suggesting there may be racial differences in the arterial structure and function, thus predisposing AA to higher risk for CVD (Kappus et al., 2017). Further, among young healthy adults, AA have been identified as having higher circulating concentrations of inflammatory cytokines (Feairheller et al., 2011; Paalani et al., 2011) as compared to W when measured at rest.

Even among young healthy adults, augmented systemic inflammation contributes to transient large artery stiffness and vascular dysfunction (Bhagat et al., 1996; Hingorani et al., 2000; Lind et al., 2012; Vlachopoulos et al., 2005). Measures of blood pressure, arterial stiffness and indices of pulse wave morphology can be used to predict the structural and functional integrity of the vasculature and provide insight into CVD risk when compared at rest and in response to an inflammatory stimulus (Mozos et al., 2017; Townsend et al., 2015).

Indeed, disparate indices of arterial structure and function between AA and W have been observed following physiological stimuli as well, such as an acute mental stress test, or cold pressor task – even when resting measures were similar between racial groups (Cardillo et al., 1998; Light et al., 1987). However, there is a lack of data regarding the effects of an acute inflammatory stimulus on the responses of central and local arterial stiffness, blood pressure and indices of pulse wave morphology between young, healthy AA and W individuals. Results from this study will provide a unique and valuable understanding of how the structure and functioning of the arterial tree adapts to a ‘physiological disturbance’, and thus may provide a basis for predicting how cumulative or chronic stressors may influence vascular health among AA and W adults.

In this context, the aim of this study was to evaluate measures of central and peripheral blood pressure, central and local arterial stiffness, and indices of pulse wave morphology in young healthy AA and W individuals in response to an acute inflammatory stimulus (influenza vaccine). The influenza vaccine was chosen as the stimulus for acute inflammation for its feasibility of administration in a young adult population, and known contribution as a mild, exogenous, inflammatory stimulus, with increases in circulating cytokine interleukin‐6 (IL‐6) activity at or around 24 h post‐vaccine administration (Kuhlman et al., 2018; Radin et al., 2021; Tsai et al., 2005).

We hypothesized that at rest, young healthy AA and W individuals would have similar central and peripheral BP, central and local arterial stiffness, and indices of pulse wave morphology. However, we hypothesized that in response to the acute inflammatory stimulus, the young healthy AA individuals would have higher central and peripheral BP, central and local arterial stiffness, and indices of the BP waveform as compared to their W counterparts.

## 2 METHODS

### 2.1 Ethical approval

All experimental procedures were approved by the Institutional Review Board at the University of Maryland, College Park on 30 August 2018 (reference number: 1292196) and complied with the most recent provisions of the *Declaration of Helsinki* (except for registration in a database). All participants provided written informed consent prior to taking part in the study.

### 2.2 Participants

A total of 29 young healthy individuals completed the study. To be included, participants were asked to confirm that they self‐identified as AA or W, were between the ages of 18 and 39, had a body mass index (BMI) <30 kg/m^2^, a systolic blood pressure (SBP) <140 mmHg, a diastolic blood pressure (DBP) <90 mmHg and were non‐users of tobacco or nicotine products. Individuals indicating the use of prescription medication for pre‐existing or current cardiovascular, metabolic and/or inflammatory diseases or conditions were excluded from the study. Additionally, participants taking antibiotics or prescription pain medications within 2 weeks of visit 1 (screening) to the laboratory, or any time during the experimental period were excluded from participation. Female participants were then asked to indicate whether they were naturally menstruating or current users of oral contraceptive pills (OCPs). Those reporting current use of OCPs confirmed at least 6 months of consistent use of the same brand and formulation. Naturally menstruating female participants, who reported not having used OCPs or other types of hormonal contraception within at least 6 months, reported regularity of their menstrual cycle. Regular menstruation was defined by researchers in accordance with the International Federation of Gynecology and Obstetrics (FIGO) recommendations on terminologies and definitions for normal and abnormal uterine bleeding (Munro et al., 2018). By these standards, normal menstruation includes monthly regularity of menses with a variance of ±4 days, frequency of menses occurring every 24–38 days, and duration of ≤8 days of menstrual flow (Munro et al., 2018). Female participants who reported irregular menstruation, amenorrhea or possible pregnancy were excluded from the study. All participants were preliminarily screened for influenza vaccine eligibility according to the 2018–2019 guidelines set by the Centers for Disease Control and Prevention and were secondarily screened by the administering registered nurse at the University of Maryland, College Park University Health Centre.

### 2.3 Study design

Participants reported to the laboratory for a total of four separate visits. Visit 1 (screening) involved eligibility screening and informed consent. Prior to the baseline (BL), 24 h (24H) and 48 h (48H) visits, participants were asked to refrain from exercise, caffeine, food and drinks other than water for ≥12 h, anti‐inflammatory medications (i.e., NSAIDs) for ≥24 h, recreational drug use for ≥48 h, and use of supplements for ≥72 h. For the 24H and 48H visits, female participants were scheduled during the early‐follicular phase of their menstrual cycle or placebo pill phase of OCP cycle to account for any impact of hormone fluctuation on measures of inflammation, haemodynamics or arterial stiffness.

### 2.4 Visit 1 (Screening)

#### 2.4.1 Resting blood pressure

Following ≥10 min of seated rest, brachial systolic and diastolic BP (bSBP and bDBP, respectively) were measured on the right arm using an automated oscillometric device (Omron Corp., Kyoto, Japan). A second set of bSBP and bDBP measurements were collected after 1 min of rest. If the two obtained bSBP and bDBP values differed by ≥5 mmHg, a third measurement was taken. An average of the two closest bSBP values was recorded and utilized for analysis. Resting heart rate was measured using a heart rate monitor (Polar Electro, Woodbury, NY, USA).

#### 2.4.2 Anthropometrics

Height and weight of participants were collected on visit 1 (screening) using a stadiometer and beam balance. Body mass index (BMI) was calculated as weight (kg) divided by height (m) squared.

### 2.5 Baseline (BL) visit

#### 2.5.1 Blood collection

Following ≥10 min of rest, 10 mL of blood was obtained from the participants’ antecubital vein and collected into serum‐separator tubes. The serum sat to clot at room temperature for 45 min, then was spun in a centrifuge at 1500 *g* for 15 min at −4°C. After centrifugation, samples were pipetted into 50 μL aliquots and stored in a −80°C freezer until analysis.

#### 2.5.2 Interleukin‐6

Commercially available high‐sensitivity enzyme‐linked immunosorbent assay (ELISA, sensitivity: 0.7 pg/ml) kits were used to measure the concentrations of serum IL‐6 according to the manufacturer's instructions (R&D Systems, Minneapolis, MN, USA). Participants’ serum samples were assayed in duplicate. Absorbances were measured using a spectrophotometer (Synergy H1 Hybrid Reader; BioTek, Winooski, VT, USA) at 450 nm. Averages of the duplicate samples at baseline (BL), 24 h post‐induced inflammation (24H), and 48 h post‐induced inflammation (48H) were recorded and used to calculate change values from BL. Changes in IL‐6 from BL (ΔIL‐6) are reported as ‘24H‐BL’ and ‘48‐BL,’ respectively.

#### 2.5.3 Peripheral and central blood pressure

Following ≥10 min of supine rest, BP waveforms were obtained from an oscillometric cuff placed snugly around participant's right upper arm (SphygmoCor XCEL, AtCor Medical, Sydney, Australia). The SphygmoCor XCEL software's validated, computerized transfer function reconstructs the brachial BP (bBP) waveforms into central aortic BP (aBP) waveforms – providing measures of brachial and aortic systolic (bSBP, aSBP), diastolic (bDBP, aDBP) and mean arterial (bMAP, aMAP) pressures, respectively.

#### 2.5.4 Indices of pressure waveforms

Central pressure waves generated by the SphygmoCor XCEL were used by the software's pulse wave analysis (PWA) function to generate the augmentation index (AIx). AIx is a composite index reflecting the influence of arterial stiffening in the large arteries, peripheral resistance in the medium and small arteries, and the reflected waveform to the heart on the central BP (Heusinkveld et al., 2019). The AIx is calculated as:

(1) $$\mathrm{AIx}\;\left(\%\right)=\frac{\left(P_{2}-P_{1}\right)}{\left(\mathrm{SBP}\;-\;\mathrm{DBP}\right)}\times 100$$

where *P*_1_ and *P*_2_ are the first and second peaks of the central arterial waveform, respectively, and the SBP and DBP are the central systolic and diastolic blood pressures, respectively.

Since an individual's HR has an impact on measures of AIx (Papaioannou et al., 2019; Wilkinson et al., 2000), values are normalized to a HR of 75 beats‐per‐minute (AIx75) by the software to minimize inter‐subject variability. To calculate the aortic forward (*P*_f_) and backward (*P*_b_) pressure waveform components, wave separation analysis was performed using SphygmoCor XCEL's synthetic triangular wave function.

#### 2.5.5 Central arterial stiffness

Central arterial stiffness was assessed using carotid‐to‐femoral pulse‐wave velocity (cfPWV). Sequential recording of the pulse waves at the right common carotid artery (CCA) and at the pulse of the right femoral artery were recorded using applanation tonometry. The distance travelled by the pressure waves between the two sites was collected with a standard tape measure; distance (m) between the suprasternal notch to the pulse of the femoral artery, the pulse of the CCA to the suprasternal notch, and from the site of the femoral pulse to the placement of a thigh cuff‐based tonometer were collected and logged into the SphygmoCor XCEL software. Transit time (s) of the pressure wave from the CCA to the femoral artery was determined via automatic detection of the diastolic foot at each pressure wave and cfPWV was calculated:

(2) $$\mathrm{cfPWV}\;\left(m/s\right)=\frac{\Delta \;\mathrm{distance}}{\Delta \;time}$$

#### 2.5.6 Local carotid arterial stiffness

The right CCA was imaged ∼1 cm below the bifurcation using ultrasonography with a high‐frequency (7.5 MHz) linear array probe (Alpha 10, Aloka, Tokyo, Japan). To capture the diameter of the CCA, echo‐tracking gates were manually set to read between the tunica intima and tunica media of the anterior and posterior CCA walls. Between 6 and 10 consecutive beats were recorded and averaged to acquire a single representative waveform. Since the carotid arterial pressure‐to‐diameter relationship is thought to be linear (Sugawara et al., 2000), measures of BP obtained during PWA were input to calculate indices of carotid arterial stiffness (β‐stiffness) and elastic modulus (Ep). Measures of β‐stiffness and Ep both reflect indices of stiffness at the carotid artery, whereby higher values of β‐stiffness and Ep indicate higher carotid arterial stiffness (Oliver & Webb, 2003). While β‐stiffness emphasizes the relative change in carotid arterial diameter in response to changes in blood pressure, measures of Ep emphasize relative change in carotid arterial diameter in response to changes in pulse pressure. The following indices were calculated:

(3) $$\beta \;Stiffness\;\left(\mathrm{AU}\right)=\ln\left(\frac{P_{s}}{P_{d}}\right)\times \left(\frac{D_{s}-D_{d}}{D_{d}}\right)$$

(4) $$\mathrm{Ep}\;\left(\mathrm{kPa}\right)=\frac{\left(P_{s}-P_{d}\right)}{\left(\frac{D_{s}-D_{d}}{D_{d}}\right)}$$

where *P*_s_ and *P*_d_ are carotid arterial systolic and diastolic BPs, and *D*_s_ and *D*_d_ are carotid arterial systolic and diastolic diameters, respectively.

Because augmented systemic inflammation has been shown to contribute to transient large artery stiffness (Bhagat et al., 1996; Hingorani et al., 2000; Lind et al., 2012; Vlachopoulos et al., 2005), it was anticipated that measures of cfPWV, β‐stiffness, and Ep would be increased in response to administration of the influenza vaccine. Further, because stiffening throughout the arterial tree has been shown to amplify BP wave reflections, which have a bidirectional relationship with increases in central BP and AIx (Laurent et al., 2006), it was anticipated that the acute inflammatory stimulus would also elicit increases in the forward and backward wave components (*P*_f_ and *P*_b_).

#### 2.5.7 Vaccine administration

At the conclusion of the BL visit, participants were walked to the University of Maryland Health Care Centre, whereupon they received a standard dose of the seasonal influenza vaccine by a registered nurse.

### 2.6 The 24H and 48H post‐BL visits

The 24H and 48H visits occurred 24 h and 48 h following the BL visit, respectively. The 24H and 48H time points were chosen to study the response of the influenza vaccine based on previous studies which have quantified the inflammatory peak (as assessed via circulating IL‐6 concentration) as occurring between 1 and 3 days post‐vaccine administration (Kuhlman et al., 2018; Radin et al., 2021; Tsai et al., 2005). The procedures in the 24H and 48H visits were identical to those of the BL visit, except for the influenza vaccine administration.

### 2.7 Statistical analysis

Baseline participant characteristics obtained on visit 1 (screening) were compared between AA and W participants using an independent Student's *t*‐test. The Shapiro–Wilk test was utilized to test all datasets for normality and the ROUT method (*Q* = 1%) was used to identify outliers. Serum concentrations of IL‐6 were quantified as 24‐h and 48‐h change values (Δ) from baseline (BL) and are expressed as ‘24H‐BL’ and ‘48‐BL’. The remaining dependent variables (bSBP, bDBP, bMAP, aSBP, aDBP, aMAP, AIx, AIx75, *P*_f_, *P*_b_, cfPWV, β‐stiffness and Ep) were compared between groups (AA and W) at three separate time points (BL, 24H and 48H). To test group × time interaction effects, the dependent variables were analysed using mixed‐effects analyses to accommodate for randomly missing values. If there was a significant effect of group, *post hoc* unpaired *t*‐tests were performed to determine at which time point the variables were significantly different between AA and W. If one or both data sets being compared were not normally distributed, the Mann–Whitney test was used instead of the unpaired *t*‐test. If the mixed‐effects analyses revealed a significant effect of time, planned paired t‐tests were performed as *post hoc* tests to determine whether there were differences between BL and 24H, BL and 48H, or 24H and 48H, among AA and/or W. If one or both data sets being compared were not normally distributed, Wilcoxon's matched‐pairs signed rank test was used instead of a paired *t*‐test. Corrected effect sizes for data were calculated using Hedges's *g*:

(5)

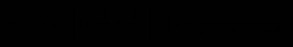




where *t* corresponds to the *t*‐value and *n* corresponds to the sample sizes (Lakens, 2013). Effects were considered small when Hedges's *g* ≤ 0.2, medium when Hedges's *g* = 0.5 and large when Hedges's *g* ≥ 0.8 (Lakens, 2013). All analyses were performed using GraphPad Prism (Version 9, GraphPad Software, Boston, MA, USA) except for effect sizes.

## 3 RESULTS

Data from all variables are reported as means ± standard deviation.

### 3.1 Participant characteristics

A total of 28 participants completed the study (AA, *n =* 11; W, *n =* 17). Participant characteristics collected during visit 1 (screening) are presented in Table 1. Independent *t*‐tests revealed no significant differences in age, BMI and bDBP between AA and W men (age: *P* = 0.805; BMI: *P* = 0.987; bDBP: *P* = 0.172; Table 1), nor between AA and W women (age: *P* = 0.356; BMI: *P* = 0.387; bDBP: *P* = 0.203; Table 1). AA women and W women also had similar bSBP on visit 1 (screening) (*P* = 0.468; Table 1). However, bSBP was significantly different on visit 1 (screening) between AA men and W men (*P* = 0.022; Table 1). Similarly, while there were no significant differences in age, BMI or bDBP between all AA and W participants when males and females were combined (age: *P* = 0.551; BMI: *P* = 0.630; bDBP: *P* = 0.860; Table 1), bSBP was significantly different between the total sample of AA and W participants, when men and women were combined into their respective racial groups (*P* = 0.037). Of the 28 participants in the study, six presented with BP values that would be considered as ‘stage 1 hypertension’, and five presented with BP values that would be considered ‘elevated BP’ according to the 2017 American College of Cardiology and American Heart Association guidelines (Whelton et al., 2018). However, these individuals were not diagnosed by a physician with hypertension at the time of their visit 1 (screening) to the laboratory. Further, none of the individuals with BPs identified as meeting the definition for ‘stage 1 hypertension’ or ‘elevated BP’ had responses to the vaccination which were physiological outliers.

**TABLE 1:** Participant characteristics.

Variable	AA participants			W participants		
	Men (*n =* 5)	Women (*n =* 6)	Total (*n =* 11)	Men (*n =* 8)	Women (*n =* 9)	Total (*n =* 17)
Age (years)	22 ± 2	21 ± 2	21 ± 2	21 ± 3	23 ± 3	22 ± 3
BMI (kg/m^2^)	24.1 ± 4.7	23.2 ± 2.2	23.6 ± 3.4	24.1 ± 2.7	22.1 ± 2.1	23.1 ± 2.5
bSBP (mmHg)	113 ± 9	111 ± 8	112 ± 8	126 ± 8^*^	114 ± 7	120 ± 10#
bDBP (mmHg)	77 ± 5	68 ± 9	72 ± 9	71 ± 9	72 ± 5	71 ± 7

* Significant difference (*P* < 0.05) in W men as compared to AA men; #significant (*P* < 0.05) difference in all W participants as compared to all AA participants. AA, African American; W, White; BMI, body mass index; bSBP, brachial systolic blood pressure; bDBP, brachial diastolic blood pressure.

### 3.2 Interleukin‐6

**FIGURE 1:**
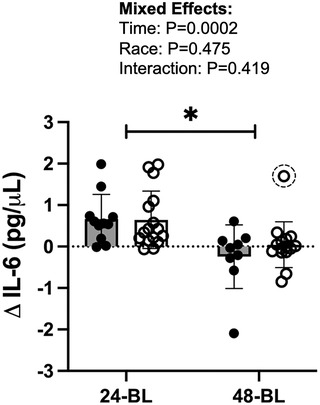
Changes (Δ) in serum concentrations of IL‐6 from baseline by 24 h (24‐BL; AA, *n =* 10; W, *n =* 16) and 48 h (48‐BL; AA, *n =* 8; W, *n =* 15; one outlier removed) post‐acute induced inflammation. Filled circles and bars represent data from African American (AA) participants. Open circles and bars represent data from White (W) participants. Dashed circles around data points indicate identified outliers. *P < 0.05.

Change in concentrations of IL‐6 from the BL visit (ΔIL‐6) at 24H (24H‐BL) and 48H (48H‐BL) were similar between AA and W participants (effect of group: *P* = 0.475). There was a significant effect of time on ΔIL‐6 (*P* = 0.0002), whereby at 24‐BL, ΔIL‐6 was significantly greater than at 48‐BL in both AA (*P* = 0.039) and W (*P* = 0.002), suggesting that the influenza vaccine successfully induced inflammation in both groups (Figure 1). Absolute values of IL‐6 concentrations have been previously published (Sapp et al., 2021).

### 3.3 Peripheral and central blood pressure

**FIGURE 2:**
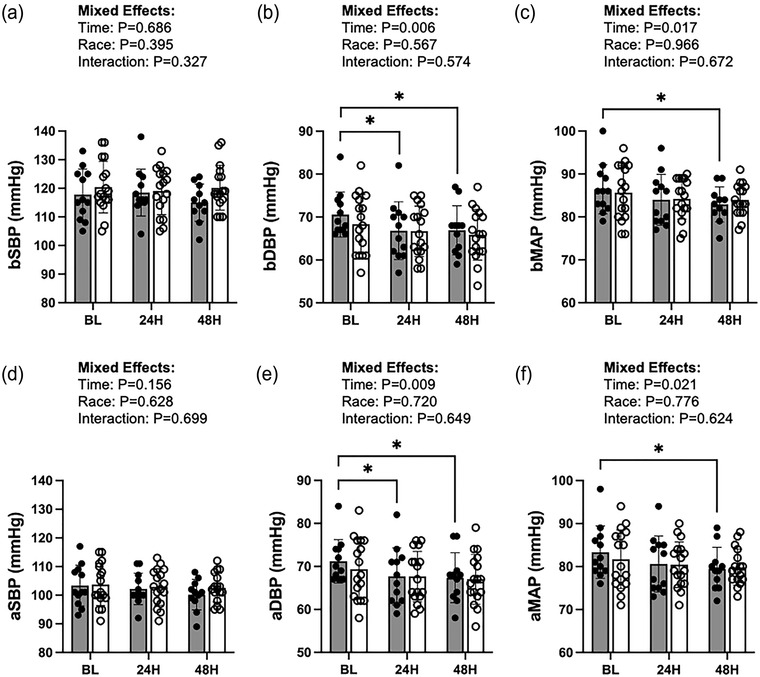
Peripheral and central haemodynamics as assessed by brachial systolic blood pressure (bSBP; a), brachial diastolic blood pressure (bDBP; b), brachial mean arterial pressure (bMAP; c), aortic systolic blood pressure (aSBP; d), aortic diastolic blood pressure (aDBP; e), and aortic mean arterial pressure (aMAP; f) at baseline (BL: AA, *n =* 12; W, *n =* 17), 24 h post‐inflammatory stimulus (24H: AA, *n =* 12; W, *n =* 17), and 48 h post‐inflammatory stimulus (48H: AA, *n =* 11; W, *n =* 17). Filled circles and bars represent individual data and means, respectively, from African American (AA) participants. Open circles and bars represent individual data and means, respectively, from White (W) participants. *P < 0.05.

There were significant effects of time on bDBP, aDBP, bMAP and aMAP (*P* = 0.008, 0.011, 0.021 and 0.027, respectively). Among AA participants, bDBP and aDBP were significantly decreased at 24H as compared to BL (*P* = 0.01, Hedges's *g* = 0.60, Figure 2b; aDBP: *P* = 0.016, Hedges's *g* = 0.58, Figure 2e); with persisting decreases at 48H as compared to BL (bDBP: *P* = 0.018, Hedges's *g* = 0.64, Figure 2b; aDBP: *P* = 0.016, Hedges's *g* = 0.35, Figure 2e). Of note, among AA participants, there were no significant differences in bDBP and aDBP between 24H and 48H (*P* = 0.896 and *P* = 0.851, respectively). Among W participants, there were no significant differences in bDBP or aDBP between any time points (*P* ≥ 0.05 for all).

Among AA, bMAP and aMAP were similar between BL and 24H (*P* = 0.070 and *P* = 0.050, respectively); however, by 48H, bMAP and aMAP were significantly lower as compared to BL (bMAP: *P* = 0.011, Hedges's *g* = 0.69; aMAP: *P* = 0.009, Hedges's *g* = 0.67). Among the AA participants, there were no significant differences in bMAP or aMAP between 24H and 48H (*P* = 0.694 and *P* = 0.596, respectively). Among W participants, there were no significant differences in bMAP or aMAP between any of the time points (*P* ≥ 0.05 for all).

### 3.4 Indices of pressure waveforms

**FIGURE 3:**
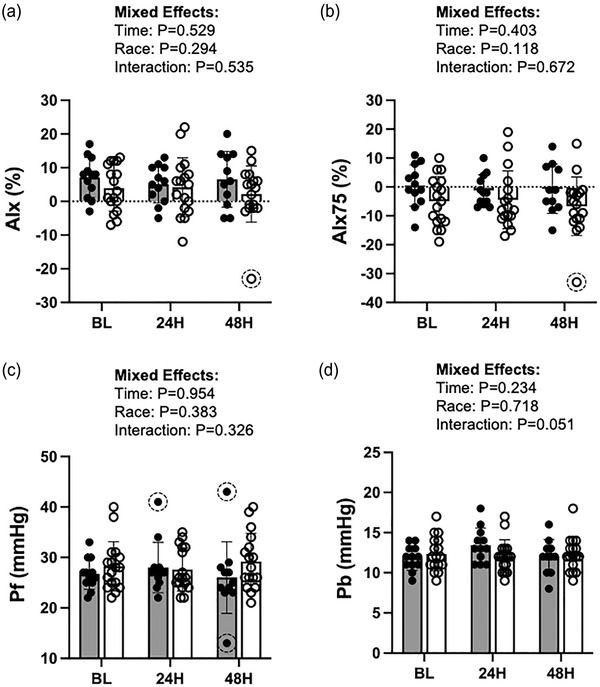
Indices of pressure waveforms, as assessed by the augmentation index (AIx; a), augmentation index standardized to a heart rate of 75 bpm (AIx75; b), forward traveling waveform (*P*_f_; c), and backward traveling waveform (*P*_b_; d) at baseline (BL: AA, *n =* 12; W, *n =* 17), 24 h post‐inflammatory stimulus (24H: AA, *n =* 12; W, *n =* 17), and 48 h post‐inflammatory stimulus (48H: AA, *n =* 11; W, *n =* 17). Filled circles and bars represent individual data and means, respectively, from African American (AA) participants. Open circles and bars represent individual data and means, respectively, from White (W) participants. Dashed circles around data points indicate identified outliers.

There was one outlier identified in the AIx (Figure 3a) and AIx75 data (Figure 3b), three outliers identified in the *P*_f_ data (Figure 3c), and none in the *P*_b_ data (Figure 3d). Regardless of whether the identified outliers were included or excluded from analyses, there were no significant effects of time post‐inflammation, racial group or interaction on AIx, AIx75, *P*_f_ or *P*_b_ (*P* ≥ 0.05 for all, Figure 3).

### 3.5 Central and local arterial stiffness

**FIGURE 4:**
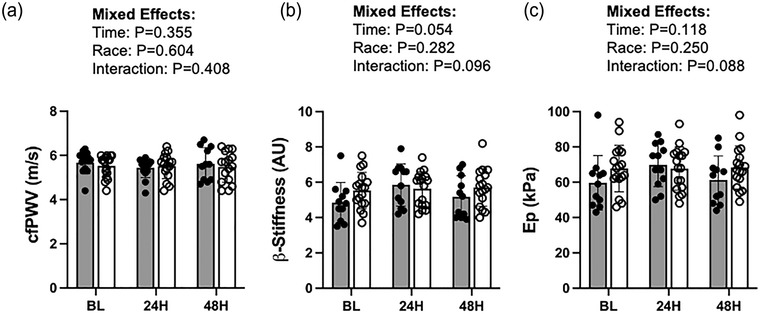
(a) Measures of central artery stiffness as assessed by carotid‐femoral pulse wave velocity (cfPWV) at baseline (BL: AA, *n =* 12; W, *n =* 17), 24 h post‐acute induced inflammation (24H: AA, *n =* 12; W, *n =* 16) and 48 h post‐acute induced inflammation (48H: AA, *n =* 11; W, *n =* 16). (b, c) Local carotid artery stiffness as assessed by β‐stiffness (b) and elastic modulus (Ep; c) at baseline (BL: AA, *n =* 11; W, *n =* 17), 24 h post‐acute induced inflammation (24H: AA, *n =* 12; W, *n =* 17) and 48 h post‐acute induced inflammation (48H: AA, *n =* 11; W, *n =* 17). Filled circles and bars represent individual data and means, respectively, from African American (AA) participants. Open circles and bars represent individual data and means, respectively, from White (W) participants.

There were no significant effects of time or racial group on cfPWV, β‐stiffness or Ep (*P* ≥ 0.05 for all, Figure 4).

## 4 DISCUSSION

Novel findings from the current study are that (1) young healthy AA and W adults have similar central and local arterial stiffness at baseline and in response to an acute inflammatory stimulus and (2) AA experience attenuated brachial and aortic DBP and MAP within 48 h following an acute inflammatory stimulus.

### 4.1 Inflammatory effects on blood pressure responses

In the present study, AA participants had significantly lower central and peripheral DBP at 24 h post‐acute inflammation as compared to BL. Further, by 48 h post‐acute inflammation, AA participants had significantly attenuated central and peripheral MAP as compared to their BL values. Contrastingly, W participants had no change in central or peripheral BP parameters at 24 h and 48 h post‐acute inflammation as compared to those at BL. Despite the significant within‐group differences in DBP and MAP, there were no between‐group differences in any of the central or peripheral BP variables at BL, 24H or 48H. Reductions in DBP and MAP have previously been observed among adults in response to an acute inflammatory stimulus (Lane‐Cordova et al., 2016; Schroeder et al., 2018); however, decreased DBP and MAP are not unanimous responses to all acute‐inflammatory models (Hingorani et al., 2000; Schroeder et al., 2019).

Reduced MAP occurring at 48 h post‐inflammation among AA in the present study may be attributable to mechanisms akin to those seen with more aggressive bouts of acute inflammation, whereby pro‐inflammatory cytokines (such as IL‐6) work to activate and overexpress inducible nitric oxide synthase (iNOS) (Chandra et al., 2006). Proliferative iNOS‐driven nitric oxide production leads to increases in peripheral vasodilatation and reductions in systemic vascular resistance which may be contributing to the observed decrease in MAP at 48 h post‐acute inflammation (Chandra et al., 2006). Indeed, baseline protein expression of iNOS has been found to be significantly higher among AA as compared to their W counterparts (Feairheller et al., 2011). Thus, acute inflammation‐induced increases in iNOS in addition to already elevated iNOS expression at baseline could explain why there were notable reductions in MAP among AA but not W individuals. However, we have reported similar brachial artery diameter and flow‐mediated dilatation (FMD) in the same AA and W individuals at 24H and 48H following acute inflammation induced by an influenza vaccine (Sapp et al., 2021). The similar arterial diameters and flow‐mediated dilatation measures between AA and W would therefore suggest that there were no noticeable decreases in peripheral vasodilatation due to acute inflammation. However, BP regulation is typically driven by microvasculature (resistance arteries) and even though flow‐mediated dilatation encompasses forearm vasculature, it is typically a measure of vascular reactivity. Therefore, it is plausible that the decreased BP responses observed in AA following acute inflammation could have been due to iNOS‐driven vasodilatation in the resistance arteries.

### 4.2 Inflammatory effects on indices of pulse wave morphology

The present study's results of unchanged AIx and AIx75 among young adults in response to an influenza vaccine are supported by findings from Schroeder et al. (2018). The current study expands on those of Schroeder et al. (2018), by suggesting that young healthy AA and W individuals experience similarly unchanged responses in AIx, AIx75, *P*_f_ and *P*_b_, to an acute inflammation. However, these findings of unchanged AIx and AIx75 specifically in response to the influenza vaccine as an acute inflammatory stimulus are in contrast with findings of decreased AIx and AIx75 in response to other methods for inducing acute inflammation, such as the typhoid vaccine (Schroeder et al., 2019; Vlachopoulos et al., 2005). One of the potential reasons for the contrasting findings could be the strength of the inflammatory stimulus or the time course measured in the present study. For example, the influenza vaccine may produce a milder inflammatory response by 24 h as compared to other methods of acute‐induced inflammation such as the typhoid vaccine. Future studies may also consider measuring vascular responses to the influenza vaccine prior to 24 h.

### 4.3 Inflammatory effects on central and local arterial stiffness

While some previous studies have suggested that an acute systemic inflammation augments central arterial stiffness (Vlachopoulos et al., 2005; Wallace et al., 2010), in the current study, young adults (AA and W) did not experience significant changes in central (cfPWV) or local (β‐stiffness or Ep) arterial stiffness in response to an acute inflammation. Additionally, data from the present study indicate that young healthy AA and W have similar central and local arterial stiffness at rest and in response to acute inflammation.

Some differences in vascular responses have been postulated to be due to stimulus severity; for example, vaccines that may result in a more potent inflammatory response such as the typhoid vaccine have shown differential effects on vascular reactions (Schroeder et al., 2019; Vlachopoulos et al., 2005). In support of this hypothesis, an existing study also using the influenza vaccine to elicit acute inflammation, reports similar findings to the present study, observing no changes in Ep, an index of local arterial stiffness, pre‐ and post‐inflammation (Schroeder et al., 2018).

### 4.4 Limitations

In hindsight, a potential limitation of the present study was our negligence in capturing indices of the participants’ social determinants of health (SDOH; economic stability, education access and quality, healthcare access and quality, neighbourhood and built environment, and social and community context). The SDOH largely influence overall health – including risk for CVD (Jilani et al., 2021). Considering the large body of evidence supporting disparate SDOH between AA and W individuals in the USA (including perceptions of racial/ethnic discrimination and institutional and individual racism), we recognize that the current study lacks the input of these variables as potential confounders to the physiological outcomes we report. Further, an individual's ethnicity (country of origin) has been linked to their physiological health. For example, foreign‐born Hispanic Americans and foreign‐born African Americans have been identified as having more favourable health profiles than US‐born Hispanics (Crimmins et al., 2007; Hummer et al., 2007; Peek et al., 2010) and US‐born African Americans (Blanas et al., 2013; Ifatunji et al., 2022) – influencing use of a term known as ‘the healthy immigrant effect’. A limitation of the present study is that we did not ask participants to provide us with information about their country of birth or immigration status. Therefore, we urge future studies investigating population‐based differences in cardiovascular physiology to incorporate measures of SDOH, as well as questionnaires that identify participants’ ethnicity and/or immigration status, especially in the context of racial differences in physiology.

### 4.5 Conclusions

Young healthy AA and W adults have similar central and local arterial stiffness at baseline and at 24 and 48 h following an acute inflammatory stimulus (via influenza vaccine); However, AA experience attenuated brachial and aortic DBP at 24 h, and attenuated brachial and aortic MAP at 48 h post‐acute inflammatory stimulus.

## AUTHOR CONTRIBUTIONS

All experimentation for this study was performed in the Human Integrative Physiology Laboratory, housed in the School of Public Health, at The University of Maryland, College Park. Lauren E. Eagan, Catalina A. Chesney, and Sushant M. Ranadive designed the research; Lauren E. Eagan, Catalina A. Chesney, and Sara E. Mascone collected the data; Lauren E. Eagan analysed the data and prepared the figures; Lauren E. Eagan, Sara E. Mascone, and Sushant M. Ranadive interpreted the results; Lauren E. Eagan and Sushant M. Ranadive drafted the manuscript; all authors edited and revised the manuscript, approved the final version, and agree to be held accountable for all aspects of the work – including ensuring that questions related to the accuracy or integrity of any part of the work are appropriately investigated and resolved. All persons designated as authors qualify for authorship, with all those qualifying for authorship being listed.

## CONFLICT OF INTEREST

The authors have no competing interests to declare that are relevant to the content of this article.

## Data Availability

The data supporting findings from this study are available upon reasonable request from the corresponding author.
